# INCONSISTENT AND UNPREPARED: HOW FAMILY CAREGIVERS AND OLDER ADULTS EXPERIENCE HOSPITAL DISCHARGE

**DOI:** 10.1093/geroni/igad104.3023

**Published:** 2023-12-21

**Authors:** Kathy Gale, Leslie Ruffalo, Melinda Kavanaugh

**Affiliations:** University of Wisconsin Milwaukee, Milwaukee, Wisconsin, United States; Medical College of Wisconsin, Milwaukee, Wisconsin, United States; University of Wisconsin Milwaukee, Milwaukee, Wisconsin, United States

## Abstract

Family caregivers and older adult patients report confusion, inconsistency, and a lack of preparation for post-discharge activities after a hospital stay, leading to hospital readmissions. This multi-disciplinary research project investigated how family caregivers, older adult patients, and hospital clinical and administrative staff experienced the hospital discharge process at a midwestern hospital system. The research team brought together expertise in community-engaged research and bridged multiple specializations, including personal family caregiver experience, community service expertise, community engaged research leadership, and instruction of medical students and social work students. Together, we conducted semi-structured interviews with family caregiver, older adult patients, and hospital personnel via video conference and telephone. We analyzed the data using open coding strategies and principles of thematic analysis. Themes included the importance of the timing of discharge planning, communication between all systems and individuals included in the discharge process, a lack of clarity regarding the roles in the process, inconsistent identification of the family caregiver, conflicting priorities, and the impact of medication errors. Implications are (1) each organization within the older adult/family caregiver system must determine their role in the post discharge process including the type of services and resources that support older adults and family caregivers and (2) the current system of healthcare, public sector, and community must determine how to adapt and change to support older adults and their family caregivers during the post-discharge period.

